# Pulmonary Melioidosis Masquerading As Tuberculosis: A Case Report Presenting a Rare Medical Condition From Western India

**DOI:** 10.7759/cureus.95272

**Published:** 2025-10-23

**Authors:** Naineesh Gaikwad, Prachee A Makashir, Rohan Kelkar, Virendra Prasad Singh, Vrushali Thakar

**Affiliations:** 1 Department of Internal Medicine, Bharati Vidyapeeth (Deemed to be University) Medical College, Pune, IND; 2 Department of Microbiology, Bharati Vidyapeeth (Deemed to be University) Medical College, Pune, IND

**Keywords:** burkholderia species, cavitary lung lesion, gram-negative bacteremia, lung tuberculosis, thoracic melioidosis

## Abstract

A young adult from a rural region of Western India developed a two-week history of intermittent fever with evening temperature spikes, dry cough, and systemic symptoms, highlighting a clinical scenario frequently encountered in endemic areas. Examination revealed crackles on the left side of the chest. Investigations revealed thrombocytopenia, mild transaminitis, and bilateral pulmonary infiltrates on chest X-ray. High-resolution computed tomography of the chest showed cavitary consolidation resembling pulmonary tuberculosis (TB), but bronchoalveolar lavage was negative for TB. Despite empirical antibiotics, the patient remained febrile. On the sixth day since admission, blood culture identified *Burkholderia pseudomallei*, confirming melioidosis bacteremia. In an endemic TB setting, this case highlights the diagnostic challenge between TB and melioidosis and underlines the importance of microbiological confirmation to guide appropriate therapy.

## Introduction

Melioidosis is a potentially fatal infectious disease caused by *Burkholderia pseudomallei* that primarily affects individuals in tropical and subtropical regions. The approximate worldwide occurrence of melioidosis is 165,000 cases and 89,000 deaths annually [1]. Recent environmental and clinical studies have highlighted a notable presence of melioidosis in India. In India, most cases have been reported from the southern states [2]. The growing epidemic of diabetes, a major risk factor, may contribute to an increase in melioidosis cases in the country. Melioidosis often resembles tuberculosis (TB) and is uncommonly suspected, making awareness crucial [3]. Humans are infected through direct contact with contaminated water or soil. The disease presents with a wide range of symptoms, including fever, pain, and organ-specific complications, often mimicking other common infections [4].

The clinical spectrum of melioidosis is highly variable, ranging from localized skin infections to pneumonia, sepsis, and multiorgan abscess formation [5]. Pulmonary involvement is particularly common and may mimic TB, presenting with fever, cough, weight loss, and cavitary lesions on imaging [6]. Such overlap in clinical presentation frequently leads to misdiagnosis in regions where TB is endemic, delaying appropriate therapy and increasing morbidity and mortality [7].

Several risk factors, such as diabetes mellitus, chronic kidney disease, chronic lung disease, immunosuppressive therapy, and excessive alcohol consumption, predispose individuals to severe disease [8]. However, melioidosis can also affect immunocompetent individuals, though less frequently, accounting for up to 20% of cases [5]. This underscores the significance of our case, as it highlights pulmonary melioidosis in an individual with no underlying risk factors, masquerading as TB, in a region of India where awareness remains low. Early recognition in such atypical hosts is crucial to prevent delays in initiating appropriate therapy.

## Case presentation

A 32-year-old man, a farmer from Western Maharashtra, presented with a history of intermittent fever with evening temperature spikes for the past two weeks. He also reported a nonproductive cough, anorexia, generalized weakness, and body pain for eight days. There was no history of dysuria, arthralgia, rash, diarrhea, or weight loss. He had no prior comorbid medical conditions.

On examination, the patient had a temperature of 40.1°C, a heart rate of 110 beats per minute, a blood pressure of 124/76 mmHg, a respiratory rate of 26 breaths per minute, and an oxygen saturation of 97% on room air. On general examination, palpable lymphadenopathy was noted, and systemic examination of the chest revealed crackles in the left mammary and interscapular areas.

Initial investigations revealed thrombocytopenia (Table 1). The liver panel showed transaminitis, and inflammatory markers were raised (Table 2). These test results suggested an evolving systemic infection. Serological and antigen-based tests for common tropical infections, including typhoid fever (Typhoid IgM), dengue (NS1 antigen), malaria, rickettsial infections, and salmonellosis, were negative. Chest X-ray showed bilateral patchy infiltrates in the mid and lower zones, suggestive of infective pulmonary lesions. The patient was started on empirical intravenous ceftriaxone in view of persistent fever.

**Table 1:** Complete blood count

Test	Day 1	Day 3	Day 4	Day 6	Normal value
Hemoglobin (g/dL)	12.6	13.6	12.6	11.9	13.2-17.5
Platelet count (/mm^3^)	44,000	90,000	151,000	351,000	150,000-350,000
Total leucocyte count (/mm^3^)	9,100	13,400	12,200	16,100	5,000-11,000

**Table 2:** Kidney, liver panel, and inflammatory markers

Test	Day 1	Day 9	Normal value
Urea (mg/dL)	25	-	17.9-54.9
Creatinine (mg/dL)	0.73	-	0.73-1.18
Total bilirubin (mg/dL)	1.0	-	0.3-1.2
Aspartate aminotransferase (U/L)	63	59	<35
Alanine aminotransferase (U/L)	69	61	<45
Serum globulin (g/dL)	2.8	-	6.4-8.3
Serum albumin (g/dL)	3.1	-	3.5-5.2
Inflammatory markers			
Erythrocyte sedimentation rate (mm/hour)	73	98	0-20
C-reactive protein (mg/dL)	100.5	40.4	<5
Serum ferritin (ng/mL)	4,349	-	20-500

**Figure 1:**
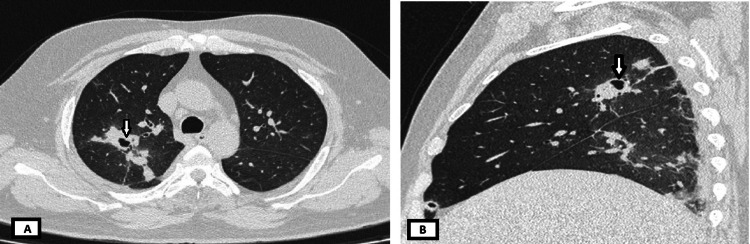
High-resolution computed tomography images of the thorax showing cavitary lesions with surrounding consolidation (A) Axial view. (B) Sagittal view

High-resolution computed tomography of the thorax revealed cavitary consolidation in the apical and anterior segments of the right upper lobe, highly suggestive of pulmonary TB (Figure 1). Bronchoscopy was subsequently performed for microbiological confirmation; however, bronchoalveolar lavage fluid culture and GeneXpert results were negative for *Mycobacterium tuberculosis*.

**Figure 2:**
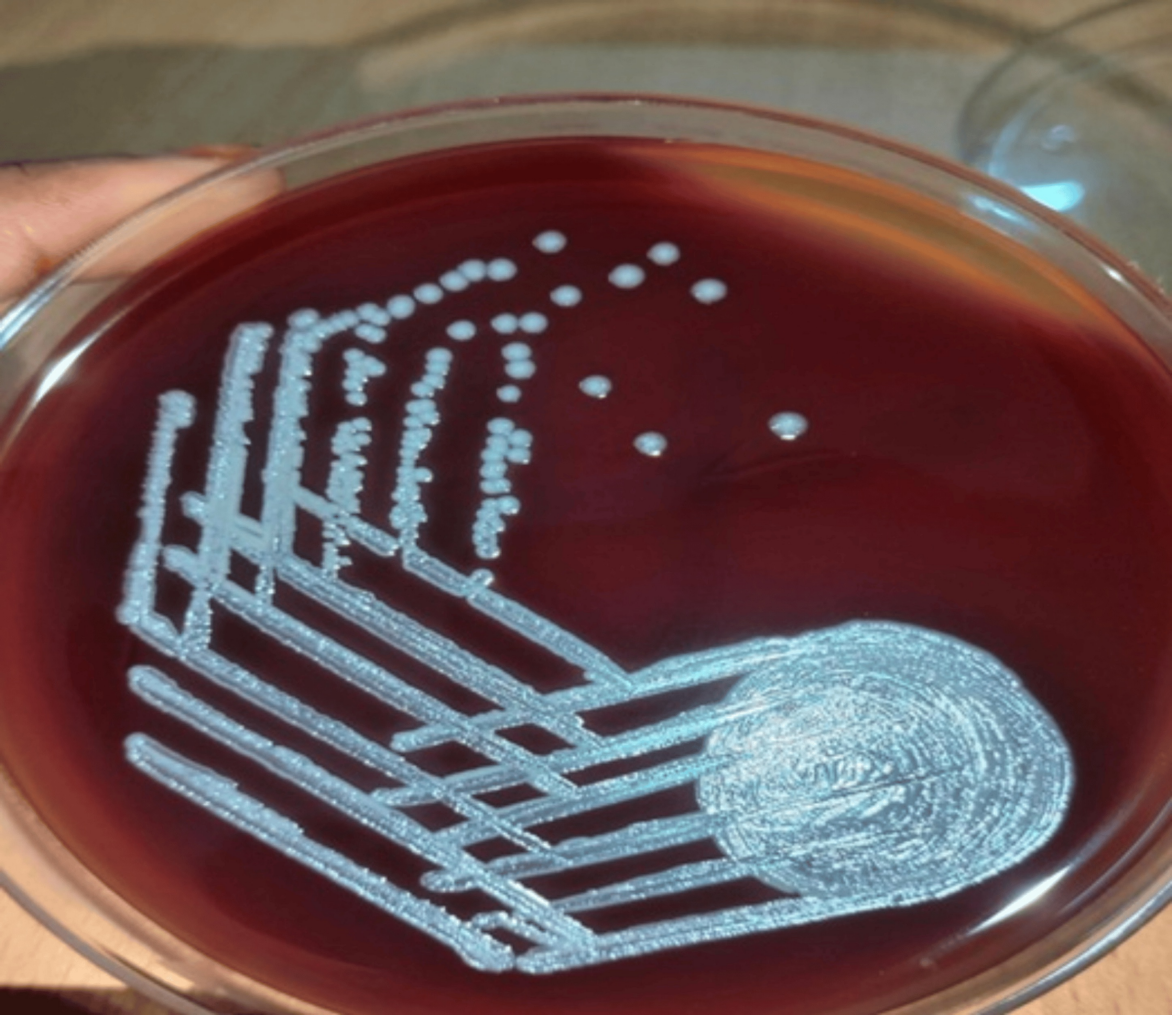
Blood culture showing growth of Burkholderia pseudomallei

On the sixth day of hospitalization, given the diagnostic uncertainty and persistent fever despite empirical therapy, a clinical dilemma arose whether to escalate to broader spectrum antibiotics. At this time, blood culture results became available and grew *B. pseudomallei*, thereby confirming the diagnosis of melioidosis bacteremia (Figure 2).

The antibiotic sensitivity report revealed sensitivity to ceftazidime, cefoperazone-sulbactam, meropenem, and trimethoprim-sulfamethoxazole (Table 3). Ceftazidime was selected for the intensive phase treatment based on proven efficacy and cost-effectiveness. The patient clinically improved after treatment with intravenous ceftazidime for two weeks. At the time of discharge, he was advised oral trimethoprim/sulfamethoxazole for three months as an eradication phase to reduce the chances of relapse.

**Table 3:** Antibiotic sensitivity pattern MIC: minimum inhibitory concentration; S: sensitive; I: intermediate; R: resistant

Antimicrobial	MIC	Interpretation
Ceftazidime	≤4	S
Piperacillin-tazobactam	≤4	R
Cefoperazone-sulbactam	≤8	S
Cefepime	16	R
Meropenem	1	S
Amikacin	≥64	R
Gentamicin	≥16	R
Ciprofloxacin	2	I
Cotrimoxazole (trimethoprim-sulfamethoxazole)	≤20	S

## Discussion

Melioidosis was first discovered by Whitmore and Krishnaswami in Myanmar and is also known as Whitmore's disease [9]. It is caused by a Gram-negative bacterium that is endemic across tropical countries, especially Southeast Asia and Northern Australia [5]. Infection is acquired by inoculation, ingestion, or inhalation of aerosols by coming in contact with contaminated soil and water [10].

Melioidosis is often underdiagnosed due to its diverse clinical manifestations and nonspecific symptoms, frequently mimicking TB, typhoid fever, or pneumonia [7,11]. Pulmonary melioidosis presents with fever, cough, and cavitary lesions, which may lead clinicians in endemic regions to empirically initiate anti-tubercular therapy, delaying appropriate management [6,7]. In our case, the patient’s persistent fever, elevated total leukocyte count, and organ-specific complications prompted a broad investigation. However, the diagnosis was not established until the blood culture yielded Burkholderia pseudomallei. This highlights the importance of considering melioidosis in the differential diagnosis of febrile illnesses in endemic regions, particularly when other common infections have been ruled out.

Although diabetes and immunocompromised states (chronic kidney disease, alcoholism, liver cirrhosis, chronic lung disease, hematological disorders, a history of splenectomy, or neutropenia) are commonly associated with melioidosis, individuals without underlying risk factors can also develop severe disease, as was illustrated in our patient [5]. Melioidosis exhibits a wide clinical spectrum, ranging from rapidly progressive, fulminant sepsis that can be fatal within days to slower, subacute illness with multiple abscesses in the lungs, liver, spleen, bones, and soft tissues. Chronic forms may persist for months to years, often presenting with indolent abscesses that mimic TB, fungal infections, or malignancy. The infection can also remain latent and asymptomatic for years, with reactivation occurring under conditions of immune compromise such as trauma, burns, diabetes, malignancy, or immunosuppressive therapy [12].

Diagnosis primarily relies on the culture of blood, pus, or other clinical specimens [13]. Molecular methods such as PCR and serology can support rapid detection but are not widely available [14]. Early recognition and prompt initiation of appropriate antimicrobial therapy are critical, as mortality rates for septicemic melioidosis can reach 50% [7]. In the intensive phase, intravenous antibiotic therapy with ceftazidime, meropenem, or imipenem is administered for 10-14 days. This is followed by a prolonged oral eradication phase with trimethoprim-sulfamethoxazole for three to six months to prevent relapse [13]. Inadequate treatment increases the risk of recurrence, emphasizing adherence to the full therapeutic regimen [15].

This case demonstrates that melioidosis should be considered in febrile patients with pulmonary lesions, even in individuals with no comorbidities, especially when TB is suspected but microbiology is negative.

## Conclusions

Melioidosis is an emerging infectious disease in India that often presents with nonspecific systemic symptoms and pulmonary findings closely resembling TB, leading to frequent misdiagnosis and inappropriate therapy. This case highlights the need for clinicians practicing in endemic regions such as Western India to maintain a high index of suspicion for melioidosis, particularly in patients with chronic febrile illness, poor response to anti-tubercular therapy, or risk factors such as diabetes and occupational exposure to soil or surface water. Early microbiological confirmation through culture and appropriate antimicrobial therapy is essential for effective management and improved outcomes. Greater clinical awareness, timely diagnosis, and inclusion of melioidosis in the differential diagnosis of TB-like presentations are critical steps toward reducing morbidity and mortality associated with this underrecognized infection.
